# A new serotyping method of *S. pneumoniae* using an automated microarray-based assay

**DOI:** 10.1186/1753-6561-5-S6-O28

**Published:** 2011-06-29

**Authors:** A Gervaix, J Corbeil, F Raymond

**Affiliations:** 1 Enfant et Adolescent, Hoptitaux Universitaires de Genève, Genève, Switzerland; 2 Medical Genomics, Laval University, Québec, Canada

## Introduction / objectives

Serotype replacement is a major concern following the introduction of polysaccharide-conjugated vaccine against *S. pneumoniae* and requires a close monitoring in the population. Antibody-based serotyping methods are expensive, semi quantitative, cross-reactions are common and a significant number of isolates cannot be typed. Multiplex PCR-based assays have been developed but quantification of PCR products remains problematic. To address these issues a novel PCR-based automated microarray assay was developed and tested on clinical samples.

## Methods

Autolysin, pneumolysin and eight other genes located in the capsular operon were first amplified using multiplex PCR. This step was followed by a tagged primer extension step targeting serotype-specific polymorphisms. The tagged primers were then assigned to a specific spot on a microarray, and processed and scanned in an ISO-certified automated molecular diagnostic system, using a confocal laser microscope. Results from the assay were exported to the analysis software/expert system that transformed genetic typing data into capsular serotype identification.

## Results

Using this new technology, 51 serotypes of *S. pneumoniae* can be precisely and uniquely identified, including the 13 types present in the new conjugate vaccine. The remaining 39 are assigned to a serogroup. Blood, CSF and nasopharyngeal samples from children with *S. pneumoniae* infection or carriage were tested and serotype was confirmed by sequence analysis. 26 different serotypes were detected and concordance between both methods was greater than 96%.

## Conclusion

This automated microarray assay is robust and could identify precise serotypes of *S. pneumoniae* directly from clinical samples. It is easy to handle and will be most useful in clinical settings and for the evaluation of serotype prevalence changes.

## Disclosure of interest

None declared.

